# Assessment of Diet Quality in Children and Adolescents with Overweight or Obesity in Greece

**DOI:** 10.3390/children10071261

**Published:** 2023-07-22

**Authors:** Odysseas Androutsos, Thomas Tsiampalis, Matina Kouvari, Maria Manou, Maria Dimopoulou, Alexandra Georgiou, Rena I. Kosti, Evangelia Charmandari

**Affiliations:** 1 Laboratory of Clinical Nutrition and Dietetics, Department of Nutrition and Dietetics, University of Thessaly, 42132 Trikala, Greece; oandroutsos@uth.gr (O.A.);; 2 Department of Nutrition and Dietetics, School of Health Science and Education, Harokopio University, 17671 Athens, Greece; 3 Discipline of Nutrition and Dietetics, Faculty of Health, University of Canberra, Canberra, ACT 2601, Australia; 4 Functional Foods and Nutrition Research (FFNR) Laboratory, University of Canberra, Canberra, ACT 2617, Australia; 5 Division of Endocrinology, Metabolism, and Diabetes, First Department of Pediatrics, Medical School, National and Kapodistrian University of Athens, ‘Aghia Sophia’ Children’s Hospital, 11527 Athens, Greece; 6 Division of Endocrinology and Metabolism, Center of Clinical, Experimental Surgery and Translational Research, Biomedical Research Foundation of the Academy of Athens, 11527 Athens, Greece

**Keywords:** obesity, diet quality, nutrition, children, adolescents

## Abstract

The adoption of healthy nutritional habits constitutes one of the most important determinants of healthy growth and development in childhood. Few studies in Greece have examined children’s diet quality using diet indices. The present study aimed to assess the diet quality of a large cohort of children and adolescents with overweight or obesity. Study participants (*n* = 1335), aged 2–18, were recruited through the Out-patient Clinic for the Prevention and Management of Overweight and Obesity in Childhood and Adolescence, Aghia Sophia Children’s Hospital, Athens, Greece. Anthropometric, socio-demographic, and behavioral data were collected using standard methods and equipment. The Diet Quality Index (DQI), which includes four subcomponents (i.e., dietary diversity, dietary quality, dietary equilibrium, and meal index), was calculated to assess each subject’s diet quality. According to the results of this study, children’s total DQI score was 63.1%. It was observed that 66.7% of the children had at least moderate diet quality (total DQI ≥ 59.34%). Boys had higher values of the total DQI and certain components of the DQI (i.e., dietary equilibrium score and meal index) compared to girls. Three out of ten children with overweight/obesity had poor diet quality (i.e., DQI ≤ 59.33). Younger children (2–5 years old) were found to have the lowest values of dietary equilibrium compared to older children (6–9 and 12–18 years old). Moreover, boys had higher values of the total DQI score and of specific components of this index (i.e., dietary equilibrium and meal index) compared to girls. Children living in urban areas had higher values in the dietary quality score compared to those living in rural areas. Children with overweight had higher values of the dietary quality score and the total DQI score compared to children with obesity. The present study highlighted that children and adolescents with overweight or obesity have poor diet quality. Multilevel and higher intensity interventions should be designed specifically for this group to achieve tangible outcomes.

## 1. Introduction

According to the World Health Organization (WHO), it is estimated that 39 million children < 5 years old and more than 340 million aged 5–19 globally are overweight or obese [1]. Very recently, the WHO revealed that overweight and obesity affect 1 in 3 school-aged children, while the prevalence in adolescence is reduced to 1 in 4 [1]. Even though it is a country bordering the Mediterranean Sea, Greece presents alarmingly high childhood obesity rates [2,3]. Based on this WHO latest report, among all European countries, Greece is on the shortlist with regards to the rates of childhood overweight and obesity [1]; 41% of children 5–9 years old are overweight, while 17.8% are obese, with the respective trends for adolescence being around 6% lower (i.e., 35.3% and 11.7%, respectively) [1]. One of the most important determinants of childhood obesity is the maintenance of a positive energy balance over the long term, which includes higher energy intake compared to energy expenditure over time. The adoption of unhealthy dietary habits may contribute to excessive caloric intake, however, in contrast, it may also increase the risk of poor diet quality and insufficient dietary intake of essential micronutrients.

Assessing children’s diet quality allows the early identification of deviations from the age-specific recommended intakes of foods and nutrients, and therefore it is important to safeguard their optimum growth, development, and health status. Dietary quality can be defined based on different criteria (e.g., variety, frequency, quantity of foods and/or nutrients consumed compared to national recommendations, etc.). Existing literature uses various methods to define diet quality, either using the a priori approach through dietary indices such as the KIDMED score [4,5] or following the a posteriori approach in which unsupervised factor analysis is used to define dietary patterns that are associated with health outcomes such as obesity [4,6]. In all cases, poor dietary quality and increased adherence to unhealthy dietary patterns have been associated with impaired weight status in the early stages of life. The association between diet quality and obesity in childhood and adolescence is complicated. A direct association could be hypothesized to be attributed to energy imbalance, while indirect associations and paths may also exist. The current literature shows that the positive association between poor diet quality and obesity is evident in vulnerable groups, including individuals of lower socioeconomic status [5,7,8]. On the other hand, other factors like age—from preschool and school age to adolescence—sex, or family status may influence this association, creating different patterns as well as different target groups from a policy making perspective.

To date, most studies have examined behaviors, lifestyles, feeding practices, and dietary habits in children and adolescents with impaired weight status using normal weight as a reference group. However, this approach does not allow a deeper comprehension of the specificities that may differentiate the profile of a child or adolescent with an overweight status (i.e., at risk of obesity) compared with a child or adolescent with a body mass index (BMI) in the zone of obesity. On the other hand, indexes assessing the overall quality of dietary habits in early life stages have been previously used in relation to impaired health conditions such as increased body weight [9,10,11]. The Diet Quality Index (DQI) stands among the very few indexes that depict different features of the diet with validation and reproducibility testing in this target group [12]. Thereby, the aim of the present study was to assess the diet quality using the DQI in a large sample of children with overweight or obesity living in Greece and to explore potential differences according to family socio-demographic characteristics and the children’s weight status. We made the hypothesis that different behavioral patterns in relation to nutrition exist among children and adolescents with impaired weight status, which is influenced not only by the weight itself but also by various demographic and environmental factors.

## 2. Materials and Methods

### 2.1. Study Design and Participants

The methodology of the current cross-sectional study is described elsewhere [13]. In brief, it was conducted from October 2014–March 2017. Study participants were children 2–18 years old who were overweight or obese and visited a weight-management outpatient clinic at the Aghia Sophia Children’s Hospital, Athens, Greece.

All subjects signed a written, informed consent form prior to their participation in the study. The study was conducted according to the Declaration of Helsinki and the conventions of the Council of Europe on human rights and biomedicine and was approved by the Committee on the Ethics of Human Research of Aghia Sophia Children’s Hospital (Approval Number: EB-PASCH-MoM: 28 November 2013, Re: 10290-14 May 2013).

### 2.2. Procedure

Children’s dietary and socio-demographic data were collected by trained researchers who conducted an interview with their parents in the Endocrine Unit of the Aghia Sophia Children’s Hospital. Children’s body weight and height were measured with standard equipment, and the International Obesity Task Force (IOTF) criteria were used to categorize children’s weight status [14]. Cases of “normal-weight” children were excluded from the current study.

### 2.3. Instruments and Variables

Socio-demographic data

Information on the children’s date of birth, gender, and region of residence was reported by the parents. The family’s region of residence was dichotomized into “urban” and “rural” regions.

Lifestyle data

Parents reported their child’s food and beverage consumption over the past 12-months via a food-frequency questionnaire (FFQ), which was previously validated in the Greek population [15]. The FFQ included 37 different foods and beverages and focused on the frequency (possible responses: “never or less than once per month”, “1–3 days per month,” “1 day per week”, “2–4 days per week”, “5–6 days per week”, and “every day”) and the portion size (possible responses in grams or ml per day, varying between the food/beverage items) for each of these foods/beverages. To facilitate the process of portion size estimation, parents were provided with a list of common standard measures as examples for each food or beverage category (e.g., 1 rusk = 10 g). The average mean intake was calculated separately for each food and beverage by multiplying the frequency of consumption (in days) with the portion size and dividing them by seven.

Children’s meal frequencies were also reported in the FFQ. More specifically, parents were asked to report the frequency of the consumption of their children for each of the three main meals (breakfast, lunch, and dinner) by selecting one of the following categories: “(almost) never”, “1–3 times a month”, “1 day a week”, “2–4 days a week”, “5–6 days a week”, and “every day”. Children were categorized as “daily breakfast eaters” (i.e. if they consumed breakfast every morning of the week) or “breakfast skippers” (i.e., if they did not consume breakfast every day of the week).

To assess children’s diet quality, the validated “Diet Quality Index” (DQI) was used. This index evaluates different components of the diet against the Flemish food-based guidelines [12]. In more detail, DQI evaluates different subcomponents: dietary diversity, dietary quality, dietary equilibrium, and meal index. “Dietary diversity” reflects the daily consumption of at least one item from each of the eight food groups recommended by the Flemish guidelines [12]. To evaluate subjects’ “dietary diversity,” the foods from the list of 37 food items were grouped into the eight food groups, their frequency of consumption was summed, and within each food group subjects were dichotomized to “1” (if they consumed ≥ 1 time/day) or “0” (if they consumed < 1 time/day). After calculating the diversity score for each of the food groups, diversity scores were summed, divided by 8 (i.e., the number of food groups), and multiplied by 100. The next subcomponent of DQI, i.e., “dietary quality”, was estimated according to the energy and nutrient density of each food item. More specifically, the “rest group”, i.e., foods that are restricted based on the Flemish guidelines [16] (e.g., sweet or salty snacks, soft drinks), were scored with “−1”; recommended foods, which are categorized as “preferred foods” in the Flemish food triangle (e.g., fresh fruit, cereal/brown bread), were scored with “1”; and those in the group that is recommended to be consumed in moderation (e.g., white bread) were scored with “0”. Then, each score was multiplied by the average daily consumption (g or ml) of the corresponding food, and all results were summed, divided by the total amount of food consumed (g or mL), and multiplied by 100. For the third subcomponent of DQI, “dietary equilibrium,” the consumption of each food group (calculated as the sum of the consumption of all food items included in this specific group) was compared against the Flemish recommendations so as to calculate both the adequacy as well as the moderation. For the calculation of each food group’s adequacy, the following algorithm was used [12]: The adequacy of each food group was scored with “1” when the food group’s daily intake covered the minimum recommended intake, while it was scored as (daily intake of the food group/minimum norm of the food group) when its daily intake did not cover the minimum recommended intake. As far as the food group’s moderation score is concerned, it was scored with “0” when the food group’s daily intake did not exceed the maximum recommended intake, with “−1” when the food group’s daily intake was twice as many or more than the maximum recommended intake, and with [(maximum norm—daily intake of the food group)/maximum norm] when the food group’s daily intake exceeded the maximum recommended intake. The total equilibrium score was the sum of all the adequacy and moderation scores divided by the total number of food groups and multiplied by 100. It is noted that in the calculation of the total equilibrium score, an additional food group was considered, namely snacks. The fourth subcomponent of DQI, “meal index,” focused on meals and was calculated by dividing the average number of times that children consumed the three main meals (i.e., breakfast, lunch, and dinner) by 3 and then multiplying by 100. The total DQI was calculated according to the following equation: *Total DQI = (dietary diversity score + dietary quality score + dietary equilibrium score + meal index)*/4. Due to the lack of national thresholds, participants were categorized using the total-DQI tertiles. Parents also reported other lifestyle behaviors of their children via a questionnaire. More specifically, they reported their child’s frequency of participation in sports (possible answers: “1 time/week”, “2 times/week”, “3 times/week”, or “>3 times/week”).

### 2.4. Statistical Analyses

Continuous characteristics are presented as mean [Standard deviation (SD)], and categorical characteristics as relative frequencies (%). The One-way Analysis of Variance (ANOVA) was used in order to investigate the association between the diet quality indices and the age group (2–5, 6–9, 10–12, 13–18 years old) in which children belong, while the independent *t*-test was performed in order to examine the association of the diet quality indices with the children’s gender (boys, girls), residence (urban, rural), and weight status (overweight, obese). A Bonferroni correction was applied in order to test the pairwise differences between the different age groups in the case of a statistically significant ANOVA result. The normality of the diet quality indices’ distribution was tested through the creation of the respective graphs (histograms, PP- plots, and QQ- plots) and the implementation of the respective statistical tests (Shapiro-Wilk test). All statistical analyses were performed using SPSS software version 29.0 (IBM Corp., Armonk, NY, USA), and the significance level was set at a = 0.05. Finally, after checking the questionnaires’ completeness for the needs of the present work, the final working sample for the analyses was n = 1335 overweight/obese children, as those with missing data on the frequency and portion size for all 37 food items were excluded from the present study.

## 3. Results

### 3.1. Demographic, Anthropometric, and Lifestyle Characteristics

The children’s characteristics are presented in Table 1. As presented, 49.6% of the participating children were aged 6–9 years old, and almost half of the children (51.5%) were girls. At least 6 out of 10 children were obese (65.3%), while with regards to their physical activity, the vast majority of the children (68.4%) were having sports activities at least three times per week.

### 3.2. Diet Quality Indices

The weekly consumption of the food groups that were used to calculate the diet quality score is presented per weight category in Table 2. As presented in Table 1, the total sample of the children had a mean dietary diversity score of 47.8%, a mean dietary quality score of 68.9%, a mean dietary equilibrium score of 40.2%, and a mean meal index score of 86.3%. With regards to the total DQI score, the participating children had a mean score of 63.1%, with 66.7% of the children being characterized as having at least moderate diet quality (total DQI ≥ 59.34%).

#### 3.2.1. Diet Quality Indices by Children’s Age and Gender

As depicted in Table 3, a significant difference was found among the four age groups in terms of their mean values in the dietary equilibrium score (*p* < 0.001), with the children aged 2–5 years old presenting the lowest values (32.2%) when compared to the rest of the age groups, while at the same time, despite the fact that the difference was not found to be statistically significant (*p* = 0.076 < 0.10), the same age group seemed to have the lowest mean total DQI value (59.7%). With regards to the gender differences, boys were found to have significantly higher mean values in the dietary equilibrium score (Boys vs. Girls: 41.8% vs. 38.8%; *p* < 0.001), in the meal index (Boys vs. Girls: 87.5% vs. 85.2%; *p* = 0.018), as well as in the total DQI score (Boys vs. Girls: 64.1% vs. 62.1%; *p* < 0.001).

#### 3.2.2. Diet Quality Indices by Children’s Weight Status and Region of Residence

As presented in Table 4, children living in urban areas had significantly higher values in the dietary quality score when compared to those living in rural areas (Urban vs. Rural: 69.6% vs. 63.9%; *p* = 0.002), while they also had borderline significantly higher values in the total DQI score (Urban vs. Rural: 63.3% vs. 61.6%; *p* = 0.076 < 0.10). As far as their weight status is concerned, overweight children were found to have significantly higher values, both in the dietary quality score (Overweight vs. Obese: 70.5% vs. 68%; *p* = 0.024) as well as in the total DQI score (Overweight vs. Obese: 64% vs. 62.6%; *p* = 0.033), when compared to obese children.

## 4. Discussion

The present study assessed the diet quality using the DQI in a large sample of children with overweight or obesity in Greece and explored differences according to the children’s sociodemographic and anthropometric characteristics (age, gender, region of residence, and weight status). In line with our research hypothesis, according to the findings, three out of ten children with overweight/obesity had poor diet quality (i.e., DQI ≤ 59.33). The sub-group analyses revealed that within this study sample of participants with overweight/obesity: (a) younger children (2–5 years old) were found to have the lowest values of dietary equilibrium compared to older children (6–9 and 12–18 years old); (b) boys had higher values of the total DQI score and of specific components of this index (i.e., dietary equilibrium and meal index) compared to girls; (c) interestingly, children living in urban areas had higher values in the dietary quality score compared to those living in rural areas; and (d) children with overweight had higher values in the dietary quality score and the total DQI score compared to children with obesity.

Diet stands among the most investigated lifestyle factors in the field of obesity. Investigating overall diet quality instead of isolating food and nutrients is more representative of the real-life scenario in all life stages [17,18]. However, assessing overall diet in childhood and adolescence remains challenging, with different existing approaches being examined to describe associations with health outcomes such as obesity [19]. Much of the adult literature uses dietary quality scores or indices as a measure of diet quality [19]. Diet quality scores are also emerging in the pediatric literature [20], and more recently, their association with health-related outcomes in children has been reviewed [21].

Hitherto, literature—including studies from Greece—suggests that poor diet quality is associated with impaired weight status in early life stages, suggesting low fruit and vegetable intake [4], low dairy and breakfast intake [22], or overall low adherence to the Mediterranean diet [5,8,23]. To the best of our knowledge, this is one of the very few studies that exclusively examined the dietary habits of children and adolescents with impaired weight status. According to the findings of the present study, overweight children had a higher DQI compared with their obese counterparts. According to the present findings, children with obesity were found to have higher consumption of certain foods (e.g., meat and fish) compared to children who were overweight. The emotional profile of obese children and adolescents is probably accompanied by increased perceived stigma, which in turn results in maladaptive responses, including increased intake of energy-dense foods and a sedentary lifestyle, as well as behaviors such as emotional overeating and binge eating [24,25]. This probably depicts the difficulty and, at the same time, the need for implementing effective strategies to promote healthier dietary habits in these categories.

The multidimensional approach of the DQI has been scarcely applied to child and adolescent populations. To the best of our knowledge, this work stands among the very few that examine the DQI in all life stages prior to adulthood. In contrast with previous works based on data from the general population that suggest that as age increases, diet quality tends to decrease [26,27], our results report a positive relationship between child age and overall diet quality. Indeed, from toddlers to adolescents with impaired weight status, there was an increasing trend in DQI. This implies that impaired weight status early in life is primarily attributed to a poor diet in terms of energy balance, diversity, and quality. Additionally, this may come as a consequence of an obesogenic family environment, including inappropriate parental modeling and/or parental feeding practices (e.g., coercive practices) [28]. This is probably modified in later life stages, with the peak in adolescence, where individuals could modify their dietary habits due to incentives such as self-body image, better performance at sports activities, or to feel approved by their peers [29,30]. The ToyBox study included a large sample of preschool children from six European countries and used the DQI to assess preschoolers’ overall diet quality and associate it with weight status. In that study, the Greek children had the lowest total diet quality score (65.2%) [2]. This low score comes in agreement with the outcome generated here in the preschooler subgroup (Total DQI: ToyBox-study (sample 3.5–5.5 years old/all weight categories) = 65.2; present study (sample 2–5 years old/only overweight/obese): 59.7). Additionally, in this study, the meal index score achieved the highest score among all four subcomponents, which is in line with the outcomes of the present work in all age groups. In contrast with our initial hypothesis [31] that this would be the case only for younger ages, it seems students in Greece—irrespective of age—consume breakfast, lunch, and dinner on a daily basis without skipping these main meals. The present study showed that preschoolers scored lower in dietary equilibrum, followed by dietary diversity; on top of this, preschoolers presented the lowest score in dietary equilibrum compared with all the rest of the age groups. We believe that this depicts an even stronger pattern of increased consumption of food products with increased energy density. With regards to dietary diversity, this has been highly discussed in terms of overweight and obesity in early life stages, with contradicting outcomes [32]. Even if the differences between the age groups were not significant, a trend was observed here, with the score increasing from preschoolers to adolescence. Picky eating or neophobia, usually observed in toddlers, may justify this observation [33].

The sex differences that exist in the adapted dietary patterns observed in early life stages have been highly discussed, with contradicting conclusions. In the present work, even if some statistically significant trends were observed between boys and girls, the actual differences did not seem to be strong enough. In particular, girls seem to reach a slightly lower overall DQI compared to boys. This finding somehow contradicts previous studies that suggest that boys have worse dietary habits than girls in terms of limiting consumption of fruits and vegetables, whole-wheat products, and food products with low fat content [34,35]. This worse dietary pattern observed in girls was primarily driven by the lower scoring in the meal index, which implies that girls tend to reduce the number of meals they consume throughout the day. This comes in line with previous evidence suggesting that girls have a tendency to skip meals like breakfast, which may reflect greater body image or dieting concerns compared to boys [36]. On the other hand, boys may prefer less healthy foods with high energy density [37]. However, here, boys scored higher in dietary equilibrium compared with girls, even if the differences were not high enough to be interpretable. Considering the cross-sectional nature of this study, the observed—apparently contradicting the existing literature—outcomes may be a matter of retrospective epidemiology depicting differentiations in the way a boy or a girl react in the context of impaired weight status. In this context, we could make the hypothesis that overweight and obese girls—predominately those in adolescence—try to skip meals to reduce their daily calorie intake and achieve weight loss. On the other hand, boys may change their dietary habits toward less energy-dense foods.

Results from studies that include diverse ethnicities have shown that rural residency is associated with an increased prevalence of childhood obesity [38]. A recent meta-analysis with 10 studies from the United States found that in a pooled population of >74,000 children and adolescents, rural residents had a close to 30% higher obesity risk compared with their urban counterparts [39]. The mechanisms through which this observation could be explained remain unclear, yet differences in dietary intake have been noted [40]. Here, we saw significantly lower overall DQI in rural regions, which was principally influenced by the dietary quality parameter. This comes in line with a very recent report for 185 countries revealing higher dietary scores, such as the Healthy Eating Index, in urban compared with rural regions [41]. Characteristics of the rural environment that may contribute to less healthy dietary habits may include poverty and low socioeconomic status, limited access to affordable healthy food, reduced access to preventative care and nutrition education, and increased stressors in the rural environment. These factors have been suggested to result in lower consumption of fruits and vegetables and emotional overeating [42,43,44]. Parental feeding practices in rural regions may also promote children’s overeating [45].

The findings of the current study should be interpreted in light of its strengths and limitations. This is one of the very few studies that attempted to investigate the dietary profile of children and adolescents exclusively with impaired weight status, investigating any trends within the zone of overweight or obesity. Additionally, standardized protocols, methods, and tools were used to collect the data (sociodemographic, lifestyle, and anthropometric). On the other hand, due to the cross-sectional design of the study, no cause-and-effect associations could be identified, while the self-reported data (e.g., lifestyle data) may be prone to recall bias or socially desirable answers. Additionally, the survey questionnaire was answered by parents irrespective of the age of participants; this may have resulted in deviations, especially when it comes to the group of adolescents. Furthermore, no information about the intensity and duration of physical activity was recorded.

## 5. Final Considerations

The present work—albeit its observational character—underscores the vulnerability of obese children and adolescents—directly compared with children and adolescents still with impaired status yet in the range of overweight—in terms of adapting to healthy nutritional habits, implying demographic, family, and psychological factors as potential moderators or mediators. Multilevel and higher-intensity interventions should be designed specifically for obese children and adolescents to achieve tangible outcomes.

## Figures and Tables

**Table 1:** Descriptive statistics of the *n* = 1335 overweight/obese children’s characteristics participating in the study.

Demographic Characteristics	N (%)
**Age**	
5 years old	85 (6.4)
9 years old	663 (49.6)
10–12 years old	241 (18.1)
13–18 years old	346 (25.9)
**Gender**—Girls	687 (51.5)
**Region of residence**	
Urban	1186 (88.9)
Rural	149 (11.1)
**Anthropometric characteristics**	
**Weight status**	
Overweight	463 (34.7)
Obese	872 (65.3)
**Lifestyle characteristics**	
**Frequency of physical activity**	
1 time/week	77 (5.7)
2 times/week	346 (25.9)
3 times/week	538 (40.3)
>3 times/week	374 (28.1)
**Diet Quality indices**	Mean (SD)
**Dietary diversity**	47.8 (22.9)
**Dietary quality**	68.9 (19.2)
**Dietary equilibrium**	40.2 (14.4)
**Meal index**	86.3 (17.6)
**Total Diet Quality Index (DQI)**	63.1 (11.2)
**DQI categories**	N (%)
Low diet quality (DQI: 15.89–59.33)	445 (33.3)
At least moderate diet quality (DQI: 59.34–90.83)	890 (66.7)

Notes: SD = Standard Deviation; Categorization of total DQI was based on the tertiles of the total DQI, in which higher scores indicate better diet quality, with 100% meaning perfect compliance with the food-based dietary guidelines; Children’s weight status was evaluated through the age- and the sex-specific International Obesity Task Force (IOTF) Body Mass Index cut-off criteria.

**Table 2:** Weekly consumption of the food groups used in the calculation of the diet quality score, separately according to the children’s weight status.

Weekly Consumption of [Mean (SD)]:	Overweight	Obese
Beverages	6.6 (1.4)	6.5 (1.5)
Bread and cereals	4.4 (2.4)	4.5 (2.4)
Potatoes and grains	6.1 (1.6)	6.2 (1.6)
Vegetables	5.6 (1.9)	5.3 (2.1)
Fruits	4.6 (2.4)	4.8 (2.3)
Milk products	5.2 (2)	4.9 (2.2)
Cheese	1.7 (2.3)	2 (2.6)
Meat and fish	2.6 (2.1)	3 (2.3)

**Table 3:** Descriptive statistics of the diet quality indices, stratified by the children’s age and gender.

	Age Groups (years)					Gender		
	2–5	6–9	10–12	13–18	*p*-Value ^1^	Boys	Girls	*p*-Value ^2^
Dietary diversity	43.3 (23.7)	47.4 (23.1)	49.0 (21.1)	50.1 (22.2)	0.132	48.0 (22.8)	47.5 (22.9)	0.664
Dietary quality	65.9 (20.4)	69.9 (18.9)	68.8 (18.9)	68.3 (20.1)	0.388	69.8 (18.2)	68.1 (20.0)	0.092
Dietary equilibrium	32.2 (14.2) ^b,c,d^	40.0 (14.4) ^a^	41.9 (14.2) ^a^	41.0 (14.4) ^a^	<0.001	41.8 (14.3)	38.8 (14.4)	<0.001
Meal index	86.1 (18.1)	87.1 (18.1)	86.4 (16.8)	85.2 (17.5)	0.606	87.5 (17.2)	85.2 (17.9)	0.018
Total DQI	59.7 (10.5)	63.5 (11.4)	63.8 (11.5)	63.2 (10.9)	0.076	64.1 (10.8)	62.1 (11.5)	<0.001

Notes: ^1^ *p*-value was based on One-way Analysis of Variance (ANOVA); ^2^ *p*-value was based on the Independent samples *t*-test; DQI = Diet Quality Index, in which higher scores indicate better diet quality, with 100% meaning perfect compliance with the food-based dietary guidelines; Statistically significant pairwise differences between the different age groups after Bonferroni corrections, are presented with superscripts a,b,c,d.

**Table 4:** Descriptive statistics of the diet quality indices, stratified by the children’s region of residence and weight status.

	Region of Residence			Weight Categories		
	Urban	Rural	*p*-Value	Overweight	Obese	*p*-Value
Dietary diversity	47.8 (22.9)	47.5 (22.3)	0.873	48.9 (22.9)	47.1 (22.8)	0.174
Dietary quality	69.6 (18.8)	63.9 (21.7)	0.002	70.5 (18.7)	68 (19.4)	0.024
Dietary equilibrium	40.4 (14.4)	39.3 (14.5)	0.371	40 (13.7)	40.4 (14.8)	0.631
Meal index	86.2 (17.8)	87.7 (16.2)	0.325	86.7 (16.7)	86.1 (18.1)	0.497
Total DQI	63.3 (11.2)	61.6 (11.3)	0.076	64 (11.2)	62.6 (11.2)	0.033

Notes: *p*-value was based on the independent samples *t*-test; DQI = Diet Quality Index, in which higher scores indicate better diet quality, with 100% meaning perfect compliance with the food-based dietary guidelines; Children’s weight status was evaluated through the age- and the sex-specific International Obesity Task Force (IOTF) Body Mass Index cut-off criteria.

## Data Availability

The data presented in this study are available on request from the corresponding author. The data are not publicly available due to data protection issues.
